# Pretreatment methods of lignocellulosic biomass for anaerobic digestion

**DOI:** 10.1186/s13568-017-0375-4

**Published:** 2017-03-28

**Authors:** Farrukh Raza Amin, Habiba Khalid, Han Zhang, Sajid u Rahman, Ruihong Zhang, Guangqing Liu, Chang Chen

**Affiliations:** 1 College of Chemical Engineering, Beijing University of Chemical Technology, 505 Zonghe Building, 15 North 3rd Ring East Road, Beijing, 100029 China; 2 College of Life Science and Technology, Beijing University of Chemical Technology, Beijing, 100029 China; 3 Department of Biological and Agricultural Engineering, University of California, Davis, CA 95616 USA; 4 Center for Climate Research and Development (CCRD), COMSATS Institute of Information Technology, Islamabad, 44000 Pakistan

**Keywords:** Lignocellulose, Pretreatment, Microaerobic, Microbial community, Biodegradation, Biogas

## Abstract

Agricultural residues, such as lignocellulosic materials (LM), are the most attractive renewable bioenergy sources and are abundantly found in nature. Anaerobic digestion has been extensively studied for the effective utilization of LM for biogas production. Experimental investigation of physiochemical changes that occur during pretreatment is needed for developing mechanistic and effective models that can be employed for the rational design of pretreatment processes. Various-cutting edge pretreatment technologies (physical, chemical and biological) are being tested on the pilot scale. These different pretreatment methods are widely described in this paper, among them, microaerobic pretreatment (MP) has gained attention as a potential pretreatment method for the degradation of LM, which just requires a limited amount of oxygen (or air) supplied directly during the pretreatment step. MP involves microbial communities under mild conditions (temperature and pressure), uses fewer enzymes and less energy for methane production, and is probably the most promising and environmentally friendly technique in the long run. Moreover, it is technically and economically feasible to use microorganisms instead of expensive chemicals, biological enzymes or mechanical equipment. The information provided in this paper, will endow readers with the background knowledge necessary for finding a promising solution to methane production.

## Introduction

Biomass resources are readily accessible around the world as residual wastes and agricultural biomass. The most important and abundant renewable biomass resources include crop residues, such as corn straw, wheat straw and rice straw. China has abundant biomass resources, as it is one of the largest agriculture-based economies in the world. China produces approximately 216 million metric tons of corn straw per annum, and more than half of that remains unutilized (Zhong et al. 2011). Corn straw contains non-edible plant material so called lignocellulose and is mainly composed of cellulose, hemicellulose, and lignin (Jørgensen et al. 2007). Hemicellulose is present as the matrix that surrounds the cellulose skeleton, while lignin is present as an encrusting material and serves as a protective layer. All three components have covalent cross-linkages between the polysaccharides and lignin, therefore, making biomass a composite material (Binder and Raines 2010). Anaerobic digestion (AD) is a promising method for the treatment of organic solid waste and wastewater, as it combines energy recovery with waste treatment. Lately, AD has been extensively used for treating highly biodegradable wastes, such as lignocellulosic materials, animal manure, kitchen waste and municipal sewage sludge (Qiao et al. 2013).

Pretreatment is an important tool for cellulose conversion processes, and is essential to change the structure of cellulosic biomass to make cellulose more available to the enzymes that convert the carbohydrate polymers into fermentable sugars (Mosier et al. 2005). The pretreatment step is referred to as the technological bottleneck for AD bioprocesses from LM that are cost effective. At least 20% of the total production cost is represented by the pretreatment phase in all these different approaches, thereby, making it the most expensive process step (Yang and Wyman 2008).

During the pretreatment process the compact structure of lignocellulosic is disrupted and cellulose fiber is exposed. Pretreatment of the lignocellulosic material is carried out to overcome recalcitrance through the combination of chemical and structural changes to the lignin and carbohydrates (Singh et al. 2015b). Previous studies have reported different methods of pretreatment, such as biological, chemical, mechanical and thermal process, as well as their combinations, to speed substrate hydrolysis (Wagner et al. 2013). However, according to a study, these traditional methods of pretreatment are cost intensive, as additional chemicals or energy are required (Lim and Wang 2013). Much research is needed to explore methods for lowering the cost of the conversion process. The basic understanding of each step in the process with regard to subsequent commercial viability and operation is required for commercial success in transforming biomass into energy.

Previous research has reported that hydrolysis can be enhanced by introducing a limited supply of oxygen during pretreatment or directly into the anaerobic digester (Ramos and Fdz-Polanco 2013). Microaerobic pretreatment is more economical and environmentally friendly compared to the other pretreatment methods, as it only requires a limited supply of oxygen. Previous studies have shown that microaerobic treatment has the potential to reduce the formation of toxic metabolites, such as ethanol and lactic acid, as well as facilitate the formation of certain lipids, which contribute to the stability of the anaerobe cell membrane (Lim and Wang 2013).

This paper reviews the pretreatment processes used in the production of biogas from lignocellulosic materials. The objective is to identify the strengths and weaknesses of various technologies and to find a pretreatment method suitable for industrial-scale adoption. We have identified the areas that need improvement in each of the mentioned technologies. In addition, some useful information for policy makers and researchers is given.

## Pretreatment methods

### Physical pretreatment

Physical pretreatment methods, including mechanical operations, different types of irradiation and ultrasonic pretreatment, have been utilized to enhance the accessibility to hydrolysable polymers within lignocellulosic material. Among the physical pretreatments, mechanical pretreatment is widely used for waste materials, such as agricultural residues or any other crops and forestry residues.

#### Mechanical

**Fig. 1:**
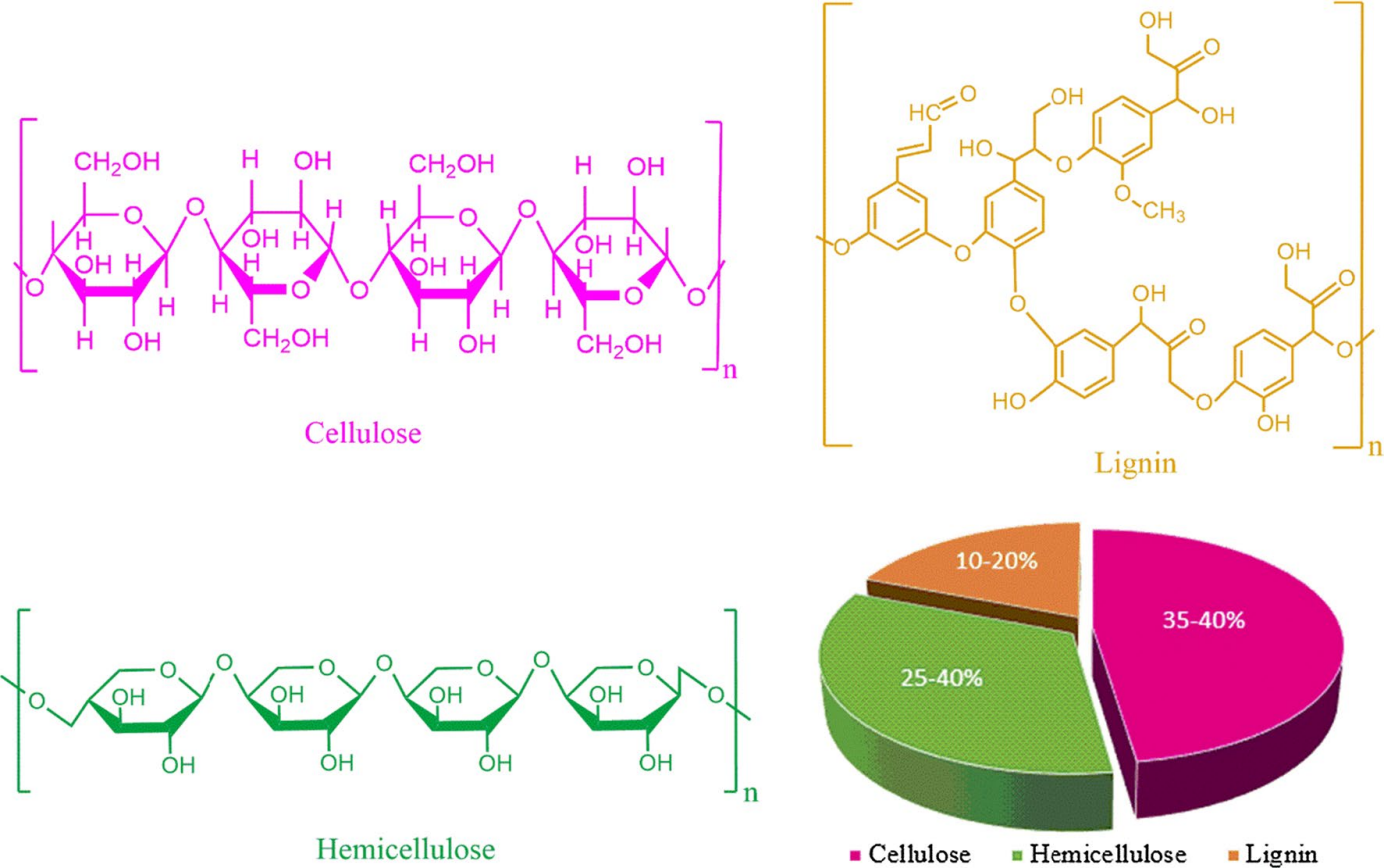
Lignocellulose composition: cellulose, hemicellulose, and lignin

Mechanical pretreatments of lignocellulosic material is an important step for improving the bioconversion affectivity, particle densification and distribution, enzymatic accessibility, and the overall transformation of lignocellulosic material into biofuels without the generation of toxic side streams (Barakat et al. 2014). This pretreatment also generates new surface area, improves flow properties, and increases the bulk density and porosity. Lignocellulosic material has an intricate composition, as shown in Fig. 1. In mechanical comminution, different mills are used to break down the lignocellulosic material and reduce the material’s crystallinity. Commonly used mills include attrition mills, ball mills, centrifugal mills, colloid mills, hammer mills, extruders, knife mills, pin mills and vibratory mills (Cheng and Timilsina 2011).

Milling reduces the crystallinity of cellulose, the substrate particle size and the degree of polymerization. The following correlation between the digestibility and structural features for wheat straw during the process of hydrolysis has been reported as shown in Eq. 1 (O’Dwyer et al. 2008).

1 $$ {\text{Digestibility}} = 2.04\left( {\text{Specific surface area}} \right)^{0.99} \times\left ({100 - {\text{Crystallinity index}}} \right) \,\times\left( {\text{Lignin content}} \right)^{{- 0.39}} $$

The process of size reduction is energy intensive. For the proper optimization and design of biomass size-reduction equipment, the mechanical properties should be well known. The feed rate of the material, initial particle size, machine variables and moisture content greatly influence the energy requirements for reducing the size of lignocellulosic material. Fine grinding requires a large amount of energy, and there is a need to maintain a balance between efficiency improvement and cost. Thus, research efforts should be made to determine the optimal size requirements of the particle size of milled biomass.

As there is no production of inhibitors, such as furfural and hydroxyl methyl furfural (HMF), milling is best suited for both ethanol and methane production. However, this technique has a high-energy requirement and is not economically viable as a pretreatment method. Considering the high-energy requirement of milling and the sky rocketing energy prices, it is likely that milling is still not an economically viable option.

### Physicochemical pretreatment

#### Steam explosion

Steam explosion (SE) is a well-known technique for the pretreatment of various biomass feedstocks. During SE pretreatment, lignocellulosic material is exposed to a high-pressure saturated steam at a temperature of 160–260 °C and a corresponding pressure of 5–50 atm for a few minutes. The pressure is gradually released, and the steam expands within the lignocellulosic matrix, causing individual fibers to separate and the cell wall structure to be disrupted (Kumar et al. 2009; Agbor et al. 2011). Acid can be added as a catalyst during steam explosion; however, the addition of acid is not mandatory. Steam pretreatment is termed as auto-hydrolysis if no exogenous acid catalyst is added to the plant biomass. However, more extensive lignin depolymerization can be achieved with 1% acid treatment.

Variables affecting the efficiency of SE include the moisture content, particle size, residence time and temperature (Talebnia et al. 2010). The particle size and composition of the starting material determine the relationship between the temperature and time (Viola et al. 2008). The cost of the overall process can be greatly reduced by using large particles. Decreasing the particle size of the material requires intensive mechanical comminution increases the production cost without significant increase in the sugar yield.

Hydrolysis and hemicellulose solubilization can be accomplished by either low temperature and long residence time (190 °C, 10 min) or high temperature and short residence time (270 °C, 1 min) (Duff and Murrayh 1996). The final selection of these two parameters, the residence time and temperature, is influenced by the pretreatment strategy as well as the physical accessibility and type of raw material.

Acetic acid is released as wood components are exposed to high-temperature steam, which further catalyze hydrolytic reactions of the constituent polymers. The loss of amorphous cellulose and hemicelluloses occurs as a consequence of these reactions (Martin-sampedro et al. 2011). The formation of formic and levulinic acids occur and can play a significant role in the pretreatment efficiency (Ramos 2003).

A commonly used parameter in steam pretreatment is the ‘severity factor’ (log R_0_), which is a measure of the severity of the pretreatment. This term combines the pretreatment temperature and the pretreatment duration in the following Eq. 2:

2 $$ {\text{log R}}_{0} {\text{ = log}}\left( {{\text{t}}*{\text{e}}^{{\left( {\frac{{({\text{T}} - 100)}}{14.75}} \right)}} } \right) $$

where log *R*_*o*_ is the severity factor (3.14–3.56 for SE) as a function of treatment time; *T* is the temperature in  °C, where 100 °C is the reference temperature at which no solubilization occurs; *t* is the residence time in (min); and 14.75 is the activation energy in the current conditions, where the process obeys first-order kinetics and the Arrhenius law (Overend and Chornet 1987).

Steam explosion is effective for the pretreatment of agricultural residues and hardwoods but less effective for softwoods, where using an acid catalyst becomes significant. The cons of SE include the incomplete destruction of the lignin-carbohydrate matrix leading to the precipitation and condensation of soluble lignin components. This destroys a fragment of the xylan in hemicellulose and generates fermentation inhibitors at higher temperatures, thus making the biomass less digestible.

#### Microwave radiation (MWR)

The electric and magnetic field components of microwaves apply forces that rapidly change in orientation at a rate of 2.4 × 10^9^ times per second (Galema 1997). MWR accelerates biological, chemical and physical processes due to heat and extensive collisions brought about by the vibration of polar molecules and ion movement (Sridar 1998). The performance of MWR is influenced by the dielectric properties of the lignocellulosic material. The ability of a material to store electromagnetic energy is measured by its dielectric constant, whereas the ability of a material to convert electromagnetic energy into heat is measured by its dielectric loss factor. The loss tangent (ratio of the dielectric loss factor to the dielectric constant) is calculated to measure the net efficiency of MWR.

The use of MWR-assisted biomass pretreatments has been studied, including (1) MWR/water, (2) MWR/alkali, (3) MWR/acid, (4) MWR/ionic liquid, (5) MWR/salt, and other combined MWR-assisted pretreatments (Xu 2015). MWR-assisted alkali pretreatment removes more hemicellulose and lignin from wheat straw in a shorter time, compared with traditional alkali pretreatment (Zhu et al. 2006b). Comparison of pretreatment with MWR/water, MWR/alkali and MWR/dilute acid showed that the maximum yield of total sugars after enzymatic pretreatment was attained from wheat straw pretreated by MWR/dilute acid (0.5% H_2_SO_4_, w/v) at 160 °C for 10 min, which was higher than that from MWR/alkali (0.1 g/g straw) at 160 °C for 10 min (604 mg total sugars/g straw) and MWR/water at 200 °C for 10 min (544 mg/g straw) (Saha et al. 2008). Microwave heating also accelerates cellulose dissolution in ionic liquids (Zhu et al. 2006a). The hydrolysis and MWR pretreatment of grass-type biomass into sugars was accomplished in one step by eliminating the hydrolysis step, making the process economically attractive (Marx et al. 2014). Currently, MWR is carried out on the lab scale, as the equipment is very small, and it is still difficult to apply in potential industrial projects; thus, it is not one of the most promising pretreatment methods.

### Chemical pretreatment

Chemical pretreatment methods are used more often than biological or physical pretreatment methods because they are more effective and enhance the biodegradation of complex materials (Zhou et al. 2012). Common chemicals used in chemical pretreatment methods for improving the AD performance of agricultural residues are sulfuric acid (H_2_SO_4_), hydrochloric acid (HCl), acetic acid (CH_3_COOH), sodium hydroxide (NaOH), potassium hydroxide (KOH), lime (Ca(OH)_2_), aqueous ammonia (NH_3_∙H_2_O), and hydrogen peroxide (H_2_O_2_) (González et al. 2005; Us and Perendeci 2012).

#### Alkali pretreatment

Alkali pretreatment involves the addition of bases to biomass, leading to an increase of internal surface by swelling, a decrease of polymerization degree and crystallinity, destruction of links between lignin and other polymers, and lignin breakdown (Badiei et al. 2014). Alkali pretreatment works better for low lignin content biomass and increasing the lignin content of biomass makes this method less effective (Sun and Cheng 2002). So the effectiveness of this pretreatment depends on the lignin content of the biomass (Mudhoo 2012). NaOH, KOH and Ca(OH)_2_ are most reported chemicals used in alkaline pretreatment, in which process conditions are relatively mild but reaction times can be long (Harmsen et al. 2010). These pretreatments are beneficial in one way or other in accomplishing the partial hydrolysis of lignocellulosic biomasses. Up to now, NaOH and KOH are the most effective alkali-treatments for improving the biomass digestibility. According to the study, the methane yield of NaOH-pretreated corn straw was found to be approximately 220 mL/g_VS_, which was 73.4% higher than that of untreated corn straw as shown in Table 1. So, NaOH pretreatment has proven to be effective to improve the digestibility and increase the methane yield. However, due to concerns over sodium discharge in the process effluent that is difficult to be recycled, may limit its application on a commercial scale (Zheng et al. 2009). Though KOH could be a solution to this problem. Considering that KOH is a strong base, KOH-pretreated anaerobic digestate is gaining more importance as a fertilizer in the agriculture sector (Jaffar et al. 2016). It has been also reported that 2.5% KOH-treated CS generates maximum methane yield of 295 mL/g_VS,_ and significantly improved 95.6% with regard to untreated CS (Li et al. 2015b). However, the high chemical loading, the toxicity to microbes, the high cost when applied in large scale, and the environmental pollution caused by the KOH is also reported (Li et al. 2015a).

**Table 1:** Different pretreatment methods and their methane yields

Pretreatment method	Pretreatment type	Pretreatment conditions	Composition changed	Gas generating capacity mL/g_VS_	Increased methane yield (%)	Observations	Reference
Physical methods	Mechanical pulverization	Pulverization, particle sizes of 33 to 6 mm	Cellulose, hemicellulose	–	11–13	Energy cost is high; particle diameter should be 6 mm for high methane yield	(Herrmann et al. 2012)
Physicochemical methods	Steam explosion	Pretreating silage straw 2.5 MPa, 90 s	Hemicellulose, lignin	334.8	56	Gas generating speed increased	(Guizhuan et al. 2012)
	Microwave radiation	Frequency 2.45 GHz, power 680 W, time 24 min	Lignin	332.3	–	Gas generating speed is fast	(Weiwei et al. 2012)
Chemical methods	H_2_SO_4_	2%, pretreated 7 days	Cellulose, hemicellulose	175.6 CH_4_	74.6	Toxic, corrosive and expensive handling	(Song et al. 2014)
	HCl	2%, pretreated 7 days	Cellulose, hemicellulose	163.4 CH_4_	62.4	Toxic, corrosive and expensive handling	(Song et al. 2014)
	CH_3_COOH	4%, pretreated 7 days	Cellulose, hemicellulose	145.1 CH_4_	44.2	Toxic, corrosive and expensive handling	(Song et al. 2014)
	NaOH	2%, pretreated 3 days	Hemicellulose, lignin	220.0 CH_4_	73.4	Toxic and hard to recycle	(Zheng et al. 2009)
	KOH	2.5%, pretreated 1 day	Hemicellulose, lignin	295.0 CH_4_	95.6	Effective but expensive	(Li et al. 2015b)
	Ca(OH)_2_	2.5%, pretreated 1 day	Hemicellulose, lignin	210.71 CH_4_	39.7	Cheap but hard to dissolve	(Li et al. 2015b)
	KOH + Ca(OH)_2_	0.5 and 2%, pretreated 1 day	Hemicellulose, lignin	271.38 CH_4_	79.9	Cheap and effective	(Li et al. 2015b)
	H_2_O_2_	3%, pretreated 7 days	Hemicellulose, lignin	216.7 CH_4_	115.4	Cheap but longer pretreatment time	(Song et al. 2014)
Biological methods	Mixed microorganism	XDC-2, pretreated for 16 days	Hemicellulose	294.9 CH_4_	87. 9	Long pretreatment time and low efficiency	(Yuan et al. 2011)
	Adding manure	Cow dung: corn straw (1:1, w/w) pretreated for 20 days	Hemicellulose	450.0	40. 7	Highly dependent on manure type	(Zhou et al. 2012)
	Microaerobic pretreatment	Pretreated up to complete O_2_ consumption by microbes	Hemicellulose, lignin	325.7 CH_4_	16.24	Efficient pretreatment and cost effective	(Fu et al. 2015d)

While, Ca(OH)_2_ might be better as it is low cost, safer, more environmental friendly, and can be easily recovered (Singh et al. 2015a). Ca(OH)_2_ has been also reported previously to enhance methane yield from lignocellulosic materials (Xiao et al. 2013). It was found that cumulative methane production of 2.5% Ca(OH)_2_-treated CS was found to be 210.71 mL/g_VS_ which was 39.7% higher than that of untreated CS (Li et al. 2015b). Nevertheless, as a weak alkali, Ca(OH)_2_ may not improve biomass digestion significantly alone.

Some reports also focused on the combinations of two or more pretreatments to increase the biodegradability and biomethane yield during anaerobic digestion processes. Such as 0.5% KOH and 2.0% Ca(OH)_2_ was comparable to the effect of 2.5% KOH, obtaining a total methane yield of 271.38 mL/g_VS_, which was 79.9% higher than that of untreated CS as shown in Table 1 (Li et al. 2015b). However, the after effects of lime in the form of precipitate, sodium salts in the form of inhibitors, and KOH as black liquor removal and relatively high price, may limit its application (Hendriks and Zeeman 2009; Li et al. 2015b). Hence, some researchers are focusing on black liquor recycling to reduce the cost as well as the pollution (Siddhu et al. 2016).

#### Acid pretreatment

In addition, CH_3_COOH, HCl and H_2_SO_4_ pretreatments have been employed for improving the AD of lignocellulosic materials (Pakarinen et al. 2011; Monlau et al. 2013). Pretreatment with acid hydrolysis (HCl, H_2_SO_4_), can result in improvement of enzymatic hydrolysis of lignocellulosic biomass, to release fermentable sugars. Acid pretreatment results in the disruption of the van der Waals forces, hydrogen bonds and covalent bonds that hold together the biomass components, which consequently causes the solubilization of hemicellulose and the reduction of cellulose (Li et al. 2010). The main reaction that occurs during acid pretreatment is the hydrolysis of hemicellulose, especially xylan, as glucomannan is more stable. Under such conditions, furfural and HMF generation can occur, because of dehydration of xylose galactose, mannose and glucose (Hendriks and Zeeman 2009). Dilute acid hydrolysis pretreatment on the other hand can achieve high reaction rates and significantly improve cellulose hydrolysis. Lignin is hardly dissolved in most cases, but is disrupted to a high degree, thus leading to increased susceptibility of the cellulose to the enzymes (Mudhoo 2012). These pretreatments are more successful with usual concentration less than 4 wt%. Acid reagents, such as H_2_SO_4_, HCl, and CH_3_COOH, at concentrations of 1, 2, and 4% (w/w) have been used for pretreatment. The biodegradation of lignocellulosic straw was effectively accomplished in all pretreatments. The straw pretreated with H_2_SO_4_ (2%) and HCl (2%) acquired the highest methane yield of 175.6 and 163.4 mL/g_VS_ among the acid pretreatments, which were 74.6 and 62.4% respectively higher than that of untreated straw, as show in Table 1 (Song et al. 2014).

For acid pretreatments, the knowledge of reaction kinetics is very important to select the suitable reactor design, configurations and operating conditions. It has been stated that hemicellulose converts to xylose by a first-order reaction with kinetic rate parameters K1 and K2 and then to furfuraldehyde and acetic acid, when biomass is exposed to a temperature higher than 180 °C (Eq. 3) (Lee et al. 1999). For the complete conversion of biomass with the high sugar and low furfural yields, hydrolysis occurs at two different stages. In the first stage, slow hydrolyzing hemicellulose at low temperature (90 °C), long retention time (50–185 min) pretreatment process with more concentrated acid (4.9–9.8%), released hemicellulosic sugars, which were then separated from biomass. While in the second stage at fast hydrolyzing hemicellulose, the remaining biomass was retreated at much higher temperature (120–130 °C) and low retention time (7–10 min) to hydrolyze cellulose to glucose. Since then, most hemicellulose hydrolysis models have been based on this reaction (Eq. 4) (Tanjore et al. 2011). However, a third variation of the basic model is the presence of an oligomeric intermediate (Eq. 5). Moreover, upon the introduction of xylo-oligomers to the kinetic analysis, the conversion of hemicellulose to soluble xylo-oligomers first occurs, which eventually converts to monomeric xylose (Jacobsen and Wyman 2000).345

Both the inhibitor formation and the hydrolysis of lignocellulose are a function of pretreatment severity, called the combined severity factor (CSF), which is influenced by the acid concentration, reaction temperature, and retention time. Chum (Chum et al. 1990) proposed an equation to calculate the CSF based on the P-factor proposed by (Overend and Chornet 1987). These relationships are indicated in Eq. 6.

6 $$ {\text{Combined Severity}}\, {\text{Factor }}\left( {\text{CSF}} \right) = \log {\text{R}}_{0} - {\text{ pH, where R}}_{0}   {\text{ = t}}^{{{\text{exp }}\left[ {\frac{{   {\text{T}}_{\text{R}} - {\text{T}}_{\text{H}} }}{14.75}} \right]}} $$

where the pH is the pH of the final slurry, t is the reaction time, T_R_ is the reaction temperature, and T_H_ is the reference temperature (100 °C).

The susceptibility of acid-pretreated biomass to cellulase treatment increases with an increase in the pretreatment severity and leads to high, nearly theoretical glucose yields. For corn straw biomass, it has been observed that with an increase in the CSF of acid pretreatment from 0.5 to 2.2, a substantially increased glucose release after enzymatic saccharification, from 32 to 57% (mass glucan/mass untreated biomass) could be achieved (Lloyd and Wyman 2005).

A feedstock pretreated with dilute acid may be slightly difficult to ferment, as fermentation inhibitors will be present. The cost of dilute acid pretreatment is higher than the other physicochemical pretreatment methods, such as AP and ammonia fiber/freeze explosion (AFEX), particularly the two-stage dilute acid pretreatment. Dilute and concentrated acids are hazardous, corrosive and toxic, and require expensive construction materials. Furthermore, acid recovery after hydrolysis leads to the secondary treatment process (Mosier et al. 2005; Kumar et al. 2009). If H_2_SO_4_ or HNO_3_ are used as chemical agents, formation of H_2_S and N_2_ due to reduction of sulphate and nitrate respectively, may cause a decrease in methane production (Hendriks and Zeeman 2009).

### Biological pretreatment

**Fig. 2:**
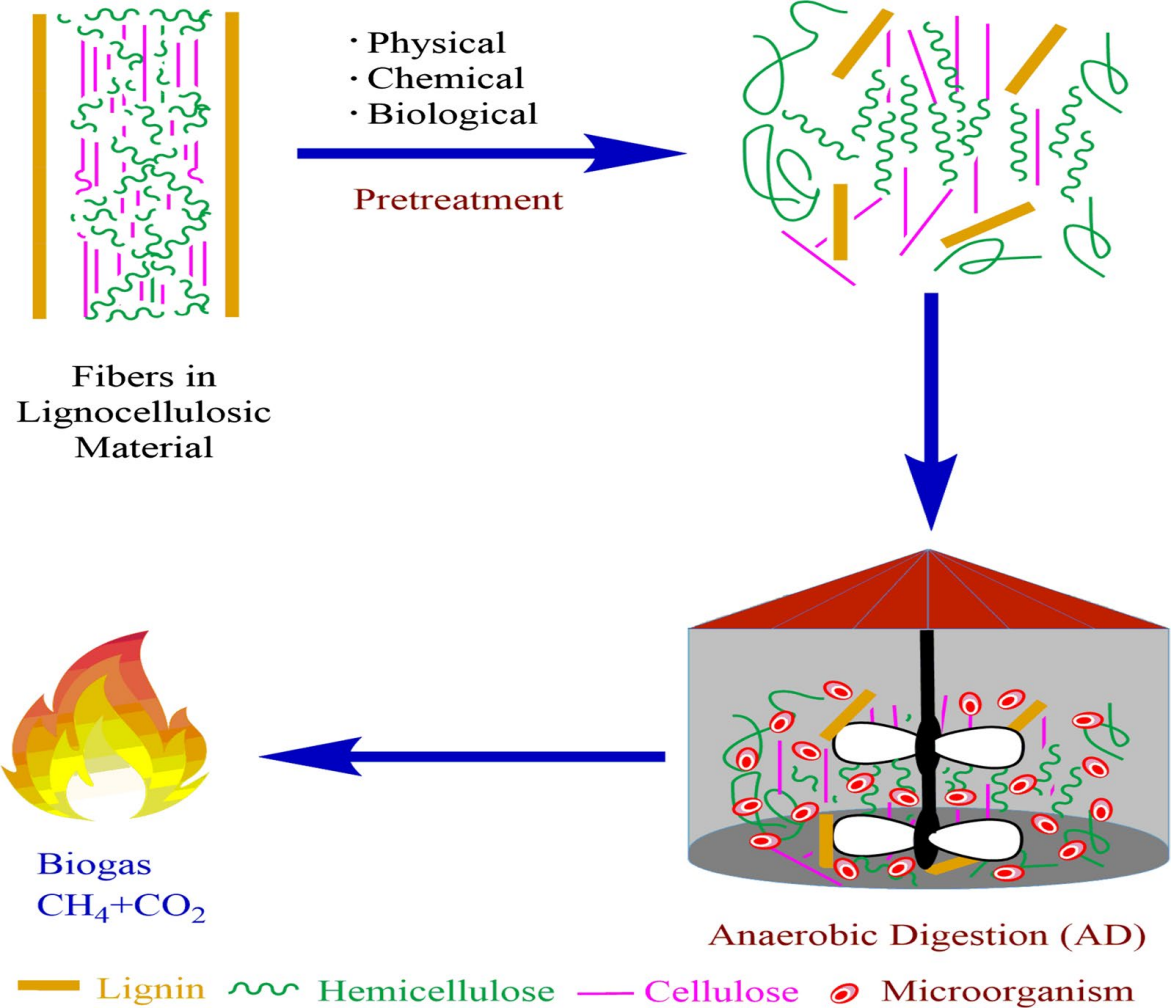
Schematic diagram of the pretreatment of lignocellulosic material for biogas production

The deconstruction of lignin structures in the cell wall using microbes and/or enzymes as catalysts is usually referred to as biological pretreatment and occurs in the first stage of hydrolysis with other pretreatment processes (Tanjore and Richard 2015). The use of cellulase enzymes for converting cellulose into oligomers and sugar monomers is termed as enzymatic saccharification and occurs in the second stage of hydrolysis. Keeping these biological processes separate is conceptually convenient, but it must be considered that many of the relevant microbes simultaneously hydrolyze cellulose and lignin to obtain carbon and energy from biomass, as shown in Fig. 2. Effective biological pretreatment requires various chemical mediators and enzymes to address biochemical and physical barriers to hydrolysis; mixtures of enzymes can work synergistically for expanding small pores and increasing access by opening the cell wall matrix (Jeremic et al. 2014).

#### Microbiological treatment

Bacteria such as *Actinomycetes*, have been observed to be effective on grasses, while fungi have gained popularity as sources of commercial plant cell wall-degrading enzymes (white-rot fungi), generating multiple cellulose-, hemicellulose- and lignin-degrading hydrolyzing enzymes. White-rot fungi have the capability to selectively metabolize low molecular weight lignin and hemicellulose while leaving cellulose relatively unaffected. *Phanerochaete chrysosporium* is the most well studied fungus for producing lignin-degrading enzymes. These aerobic bacteria are grown on biomass by utilizing solid-state fermentation technologies familiar to simple bench-scale laboratory systems and the mushroom industry (Saritha 2012).

The rate of biological pretreatment is very slow for industrial purposes. Some of the disadvantages of biological pretreatment that make it less suitable for industry include a long residence time of 10–14 days, extremely precise growth conditions, and the need for a large space to perform the biological pretreatment. Another potential disadvantage is that some fraction of the carbohydrate is consumed by the microorganisms. Biological pretreatment can be exploited as a first step, default pretreatment in combination with another pretreatment method or on its own if the biomass has a low lignin content (Agbor et al. 2011).

However, the most cost-effective and favorable treatments among these still need to be identified. Furthermore, the optimal concentrations for pretreatment not often reported. For the efficient and feasible utilization of agricultural residues, such information is essential.

#### Microaerobic pretreatment

Microaerobic pretreatment (MP) is considered to be an alternative pretreatment for the AD of corn straw in various studies. Oxygen conventionally inhibits AD. Recent studies have shown that introducing a limited supply of oxygen (or air) directly into the AD or during the pretreatment step can improve the methane yield of corn straw. The relative abundance of phylum *Firmicutes*, class *Clostridia* and order *Clostridiales*, which are associated with hydrolysis of AD, were grown under microaerobic conditions. Furthermore, the relative abundances of *Methanobacterium* and *Oxytolerant* were both doubled under microaerobic conditions. The reason for the improved AD performance may be the microbial community shifting under microaerobic conditions (Fu et al. 2016).

The amount of oxygen supplied during pretreatment is very important, as excessive oxygen inhibits the activity of methane-forming microorganisms and decreases the production of methane (Xu et al. 2014). In contrast, excessive oxygen can oxidize readily available substrates or facilitate aerobic M*ethanotrophs* to consume methane. It has been also reported that thermophilic microaerobic pretreatment (TMP) before the AD of corn straw resulted in an increase in the relative abundance of phylum *Firmicutes*, which are associated with the production of extracellular enzymes. The relative abundance of phylum *Firmicutes* (especially class *Bacilli*, order *Bacillales*) was higher under microaerobic conditions than anaerobic conditions, which enables and increase in extracellular enzymes, reducing sugar, volatile fatty acids (VFAs) and soluble chemical oxygen demand (SCOD) under microaerobic condition. Therefore, the AD of corn straw was more efficient, and more methane was produced (Fu et al. 2015b).

The influence of AP and TMP on the AD of sugarcane bagasse was studied. It was seen that both AP and TMP were efficient pretreatment methods for the AD of sugarcane bagasse. The oxygen loading during TMP is of vital importance in the maximum cumulative methane production of sugarcane bagasse and can result in better crystallinity disruption, VS removal, and methane production with less lag-phase time, whereas, AP efficiently removes lignin in addition to improving the methane production rate and technical digestion time. AP requires a large amount of chemical reagent during pretreatment, whereas TMP is a cost-effective and eco-friendlier pretreatment method for the AD of sugarcane bagasse as shown in Table 2 (Fu et al. 2015a).

**Table 2:** Microaerobic pretreatment of lignocellulosic biomass

Conditions for feedstock			Conditions for inoculum			Pretreatment process	O_2_ Conc. (mL/g_VS_)	Digestion process/Temp (°C)	Gas yield (mL/g_VS_)	Improved yield (%)	Reference
Type	TS (%)	VS (%)	Type	TS (%)	VS (%)						
Corn straw	92.4	93.4	Biogas slurry	6.64	70.62	TMP	5	Batch/37	325.7 CH_4_	16.24	(Fu et al. 2015d)
Corn straw	91.9	89.5	Active sludge	2.6	52.7	TMP	0.45/day	Batch/55	216.8 CH_4_	16.5	(Fu et al. 2016)
Corn straw	92.4	93.4	Biogas slurry	6.1	73.05	Secondary TMT	10	Batch/37	380.6 CH_4_	28.4	(Fu et al. 2015c)
Corn straw	91.25	91.8	Biogas slurry	1.25	0.75	Micro-aerobic	0.28/day	Batch/35	41.6 H_2_	43	(Li et al. 2016)
Sugarcane bagasse	29.6	96.2	Anaerobic sludge	4.6	70.6	TMP	10	Batch/37	229.6 CH_4_	29.28	(Fu et al. 2015a)

In addition to pretreatment before anaerobic digestion some researchers have suggested substrate pretreatment during the anaerobic digestion process. Secondary thermophilic microaerobic treatment (STMT) during anaerobic digestion helps in reset the digestion process by buffering the pH and increasing the microorganism activity, which provides a secondary increase in biomass degradation. This may be a successful solution to improve the low fermentation efficiency during the later stages of the anaerobic digestion process. The effect of STMT on the anaerobic digestion of corn straw improved the VS removal efficiency and afforded a higher methane yield. Similar to microaerobic pretreatment before the anaerobic digestion process, the oxygen supply in STMT during anaerobic digestion process not only reduces the concentration of toxic metabolites (e.g., ethanol, acetic acid and lactic acid) but also promotes the synthesis of certain lipids required for the stability of the anaerobe cell membrane (Fu et al. 2015c) as shown in Table 2.

Thus, TMP can be considered to be an efficient pretreatment process for AD when methane yield enhancement is a primary concern. This option has strong ability to accelerate hydrolysis, reduce the lag-phase time, and increase the methane production up to 16.24% higher than that of untreated corn digestion. The decrease in the crystallinity index, resulting from structural changes during the TMP process, may be the cause of the improvement in the methane yield of pretreated biomass as shown in Table 2 (Fu et al. 2015d).

Based upon the literature studies, it is concluded that each pretreatment method has its own merits and demerits. Although, based on feedstock types and availability of technology, the appropriate method can be selected. Among the various cutting-edge technologies, MP may be an efficient and cost effective pretreatment method that seems like a promising method and it may meet the requirements for industrial scale adoption. However, what happened during MP process is still less reported and study on whole mechanism of MP process is also lacking. Further research for technological advancement is highly recommended.

## Conclusion

Pretreatment alters the various feedstock characteristics at the fiber, fibril and micro fibril level. The extent and rate of LM hydrolysis are affected by biological pretreatment, chemical pretreatment, physical pretreatment, and its morphological characteristics. However, the most cost-effective and favorable treatments among these methods have not yet been identified. Moreover, the optimal conditions for pretreatment are rarely reported. Such information is essential for the efficient and feasible utilization of different agricultural residues. One of the potential pretreatment methods reported in different studies is microaerobic pretreatment, which is more economical and environmentally friendly. MP only requires a limited amount of oxygen (or air) supplied either during a pretreatment step or directly into the anaerobic digester. The mechanism behind microaerobic pretreatment is hydrolysis initiated by the increased facultative bacteria growth rate and enzymatic activity and the greater cellulase production under microaerobic conditions. MP is an efficient and cost-effective pretreatment method that meets most of the requirements for industrial applications, such as the formation of reactive cellulosic fiber for enzymatic attack, the avoidance of the formation of possible inhibitors to the fermenting microorganisms and hydrolytic enzymes, reduced energy demand and reduced cost of size reduction of the feedstock. Other benefits include the reduction in the cost of material for construction of the pretreatment reactor and the generation of fewer residues due to zero consumption of chemicals, all of which may make MP one of the most promising and environmentally friendly techniques in the long run. At present, researchers and policy makers are in dire need of useful information that may lead to the necessary improvements in the AD industry.

## Abbreviations

AD: anaerobic digestion
AP: alkali pretreatment
AFEX: ammonia fiber/freeze explosion
CS: corn straw
CSF: combined severity factor
HMF: hydroxyl methyl furfural
LM: lignocellulosic materials
MC: moisture contents
MP: microaerobic pretreatment
MWR: microwave radiations
SCOD: soluble chemical oxygen demand
SE: steam explosion
STMT: secondary thermophilic microaerobic treatment
TMP: thermophilic microaerobic pretreatment
TS: total solid
VFA: volatile fatty acid
VS: volatile solid
